# Gastric neuroendocrine neoplasias: manifestations and comparative outcomes

**DOI:** 10.1530/ERC-18-0582

**Published:** 2019-07-04

**Authors:** S Felder, H Jann, R Arsenic, T Denecke, V Prasad, B Knappe-Drzikova, S Maasberg, B Wiedenmann, M Pavel, A Pascher, U F Pape

**Affiliations:** 1 Medizinische Klinik mit Schwerpunkt Hepatologie und Gastroenterologie (einschl. Arbeitsbereich Stoffwechselerkrankungen), Charité – Universitätsmedizin Berlin, Campus Virchow-Klinikum, Berlin, Germany; 2 Institut für Pathologie, Charité – Universitätsmedizin Berlin, Campus Mitte, Berlin, Germany; 3 Klinik für Radiologie, Charité – Universitätsmedizin Berlin, Campus Virchow-Klinikum, Berlin, Germany; 4 Klinik für Nuklearmedizin, Charité – Universitätsmedizin Berlin, Campus Virchow-Klinikum, Berlin, Germany; 5 Klinik für Nuklearmedizin, Universitätklinikum Ulm, Ulm, Germany; 6 Innere Medizin und Gastroenterologie, Asklepios Klinik St. Georg, Asklepios Medical School, Hamburg, Germany; 7 Medizinische Klinik 1, Gastroenterologie, Pneumologie und Endokrinologie, Universitätsklinikum der Friedrich-Alexander Universität Erlangen, Erlangen, Germany; 8 Klinik für Allgemein-, Viszeral- und Transplantationschirurgie, Charité – Universitätsmedizin Berlin, Campus Virchow-Klinikum, Berlin, Germany; 9 Klinik für Allgemein-, Viszeral- und Transplantationschirurgie, Uinversitätsklinikum Münster, Münster, Germany

**Keywords:** gastric neuroendocrine neoplasia, classification, TNM, overall survival, long-term outcome

## Abstract

Although gastric neuroendocrine neoplasias (gNEN) are an orphan disease, their incidence is rising. The heterogeneous clinical course powers the ongoing discussion of the most appropriate classification system and management. Prognostic relevance of proposed classifications was retrospectively analysed in 142 patients from a single tertiary referral centre. Baseline, management and survival data were acquired for statistical analyses. The distribution according to the clinicopathological typification was gNEN-1 (*n* = 86/60.6%), gNEN-2 (*n* = 7/4.9%), gNEN-3 (*n* = 24/16.9%) and gNEN-4 (*n* = 25/17.6%), while hypergastrinemia-associated gNEN-1 and -2 were all low-grade tumours (NET-G1/2), formerly termed sporadic gNEN-3 could be subdivided into gNEN-3 with grade 1 or 2 and gNEN-4 with grade 3 (NEC-G3). During follow-up 36 patients died (25%). The mean overall survival (OS) of all gNEN was 14.2 years. The OS differed statistically significant across all subgroups with either classification system. According to UICC 2017 TNM classification, OS differed for early and advanced stages, while WHO grading indicated poorer prognosis for NEC-G3. Cox regression analysis confirmed the independent prognostic validity of either classification system for survival. Particularly careful analysis of the clinical course of gNEN-1 (ECLomas, gastric carcinoids) confirmed their mostly benign, but recurrent and extremely slowly progressive behaviour with low risk of metastasis (7%) and an efficient long-term control by repetitive endoscopic procedures. Our study provides evidence for the validity of current classifications focusing on typing, grading and staging. These are crucial tools for risk stratification, especially to differentiate gNEN-1 as well as sporadic gNET and gNEC (gNEN-3 vs -4).

## Introduction

Gastric neuroendocrine neoplasias (gNENs) are a heterogeneous subgroup of gastroenteropancreatic neuroendocrine neoplasias (GEP-NENs) derived from specialized cells of the diffuse endocrine system and were initially described as gastric carcinoids by Max Askanazy, who already noted their similarity to the carcinoid tumours detected by Oberndorfer (Oberndorfer 1907, Askanazy 1923). The incidence of GEP-NEN in general and gNEN in particular has been rising throughout the last decades, probably because of increased awareness and broader use of diagnostic modalities such as endoscopy and standardized histopathological work-up (Boyce & Thomsen 2015, Dasari *et al.* 2017). Recently published epidemiological data report a gNEN prevalence of 8.9% (5–23%) of all GEP-NEN (Delle Fave *et al.* 2016). While the incidence of gNEN is rising worldwide, there is a clear trend for improved survival (Yang *et al.* 2018). The clinical and histopathological classification of gNEN into three subtypes (Rindi *et al.* 1993, 1996) is widely accepted and proposed in guidelines by ENETS, NANETS and WHO (Rindi *et al.* 2006, Ruszniewski *et al.* 2006, Kulke *et al.* 2010). Accordingly gNEN-type 1 (gNEN-1) are associated with hypergastrinemia (HG) in chronic atrophic gastritis (CAG), while gNEN-type 2 (gNEN-2) are resulting from HG caused by Zollinger-Ellison-Syndrome (ZES) either in a sporadic gastrinoma or associated with MEN-1-syndrome. These NENs putatively arise from the ECL cells of gastric corpus and fundus mucosa and have thus also been termed ECLomas (Rindi *et al.* 1993, 1996). In contrast gNEN-type 3 (gNEN-3) appear sporadically without yet known cause. In CAG the gNEN-1-incidence ranges between 23.4 and 39.1% (Massironi *et al.* 2015, Campana *et al.* 2017). The annual incidence rate is 0.4% per 1463 person years (Vannella *et al.* 2011). Independent risk factors for the development of gNEN-1 are identified: age over 59 years, male gender, elevated chromogranin A (CgA) serum levels and histological findings of intestinal metaplasia (Campana *et al.* 2017). The long-term intake of proton pump inhibitors as independent risk factor for gNEN-1 is still under discussion, but new evidence questions this association (Soto-Solis *et al.* 2019).

Others proposed four subtypes by differentiating the sporadic gNEN-3 subgroup according to the Ki-67-labelling index-based grading in gNET-3 and gNEC-4 (Klöppel *et al.* 2007); because of supposed clinical relevance, this proposal was recently adopted by the German S2K-Guideline for Neuroendocrine Tumours (Rinke *et al.* 2018). Up to date decision making is realigned to clinicopathologic type, grade and stage of the tumour (Borch *et al.* 2005). The preconditions of an accurate treatment of patients with gNEN are a standardized histopathologic diagnosis and a commonly accepted while easily applicable classification system (Klimstra *et al.* 2010). For GEP-NEN the TNM-Staging and the Ki-67-labelling index-based grading gained wide acceptance for risk stratification due to proven prognostic relevance (Rindi *et al.* 2006, Pape *et al.* 2008*b*, Bosman *et al.* 2010). The refinement of the classifications is a constant process including the recent UICC TNM Staging update (Kim *et al.* 2016, Gospodarowicz *et al.* 2017). New findings from the SEER database propose that more than six metastatic lymph nodes in partial or total gastrectomy gNEN are an independent predictor of survival (Pak *et al.* 2019).

On the other hand, WHO stated the heterogeneous course of gNEN-1 as mostly benign but rarely as of uncertain behaviour and the best clinical management is still open to a long-standing debate (Solcia *et al.* 2000). The spectrum of recommended therapies ranged from aggressive surgery to even ‘watch and wait’ in early stages and therefore alternated in the past between presumed underestimation of the malignant potential and debilitating overtreatment (Ravizza *et al.* 2007, Massironi *et al.* 2009). Current guidelines are controversial when it comes to best treatment and follow-up strategies in gNEN-1 (Kaltsas *et al.* 2014, Delle Fave *et al.* 2016). The likelihood for the even rare formation of metastasis of gNEN-1 is directly related to size and depth of invasion (Rindi *et al.* 1996, Saund *et al.* 2011) as well as to elevated Ki-67 values and serum gastrin levels (Grozinsky-Glasberg *et al.* 2013). Recently proposed size-based risk stratification differentiates low- (<1 cm), intermediate- (1–2 cm) and high-risk groups (>2 cm) (Saund *et al.* 2011, Kaltsas *et al.* 2014). However, clinical guidance based on recent outcome data is lacking; therefore, the aim of our study was to identify prognostic factors of outcome in gNEN.

## Materials and methods

### Patient selection, data collection and processing

The medical records of 207 patients with gNEN treated at our institution between January 1992 and December 2014 were analysed retrospectively. Histopathologic confirmation of a gNEN and age above 18 years at the time of hospital admission were required for inclusion. Consent for scientific analysis of routine clinical data was obtained on hospital admission in conformity with the local ethics committee’s and data protection committee’s rules, which approve centre-based scientific analyses of data from patients treated at our institution by treating physicians as long as written general consent to scientific data analysis has been provided by each individual patient after informed consent. Patient files were reviewed systematically for date of initial diagnosis (ID), gastric localization of primary tumour, histopathologic grading and staging, clinical staging (i.e. results of resective procedures and imaging studies) and management information including treatment decisions (SF). Collected data were re-evaluated for correctness and consistency (SF, HJ, UFP). Survival data were obtained by chart review. According to the available information, the clinicopathologic typification as proposed by Klöppel *et al*., the renewed UICC TNM staging system and the WHO grading system were applied whenever possible (Rindi *et al.* 2006, Klöppel *et al.* 2007, Bosman *et al.* 2010, Delle Fave *et al.* 2016, Gospodarowicz *et al.* 2017).

### Definition of baseline data and alternative endpoints

The clinical and biological features of gastrinoma and specially the secondary character of gNEN type 2 (gNEN-2) lead to relevant methodological problems in retrospective analysis. Mainly the bias resulting from the presence of another primary (pancreatico-duodenal gastrinoma) with higher prognostic relevance was the rationale for exclusion of gNEN-2 from further outcome analysis.

Considering the pathophysiologies of gNEN, we defined baseline characteristics, primary and secondary endpoints as follows: pathophysiological, histological and clinical information available at ID were regarded as baseline information. In patients with gNEN-1, we defined an initial diagnostic period (IDP) of 6 months after date of ID and summarized all new histological and clinical data within this timeframe as baseline data. In patients with sporadic gNEN (gNEN-3 or gNEN-4), we defined the IDP as 3 months and likewise assessed all information recorded within this period as baseline data (Delle Fave *et al.* 2016). The routine follow-up approach included repetitive upper endoscopy, abdominal ultrasound, computed tomography of the abdomen at 3–6 monthly (if stable) intervals and somatostatin receptor imaging (SRI) as required by suspicion of progressive disease.

We recorded data referring to ‘last well seen’ or ‘date of death’ for overall and NEN-related survival analyses. Endpoint definition was particularly challenging in gNEN-1 because of scarce deaths. Frequently performed removal by forceps rarely determined R0-results. Consecutively the endpoint ‘recurrence’ was not accessible. Therefore, histological evidence of gNEN-1 manifestations collected after the IDP was classified as ‘evidence of persistence’ (EP). All cases with histological evidence of gNEN-1, collected after the IDP, which showed additional progress in size, number, grading or respective clinical histopathologic staging were classified as ‘progressive disease’ (PD).

### Statistical data analysis

Statistical data analysis was conducted using SPSS, version 25.0 (SPSS Inc.). The distribution of continuous variables is reported as mean, standard deviation (s.d.), standard error of the mean (s.e.m.), range and 95% confidence interval (CI). After normal distribution was assessed by Kolmogorov–Smirnov test (for two samples and adhering to the Levene’s test of equal variances) the comparisons were performed with *T*-test or with Pearson’s chi-square test and Fisher’s exact (Fiex) if necessary. Mann–Whitney *U* test was applied for categorical variables and for continuous variables if normality was not given. All tests were two sided; a *P* value of <0.05 was considered statistically significant.

Overall survival (OS), time to evidence of persistence (TTEP) and time to progressive disease (TTPD) were estimated using the Kaplan–Meier method and tested for statistical significance by log-rank testing for all gNEN. We conducted a univariate analysis of baseline characteristics, followed by a multivariate analysis of significant variables. The relative hazard for EP and PD in gNEN-1 was calculated. Especially the relative risk of OS and NEN-related survival depending on typing, staging and grading was compared with the lowest risk group and analysed using an age- and gender-adjusted Cox proportional hazards model (Rindi *et al.* 2006, Klöppel *et al.* 2007, Bosman *et al.* 2010, Delle Fave *et al.* 2016, Gospodarowicz *et al.* 2017).

## Results

### Cohort characteristics

In total, 142 patients with gNEN, who were treated at our institution between January 1992 and December 2014, met inclusion criteria and were analysed retrospectively. According to the clinicopathologic typification, 86 patients (60.5%) had CAG-associated gNEN-1, 7 (5%) had ZES-associated gNEN-2 and 49 (34.5%) had sporadic gNEN-3 (Delle Fave *et al.* 2016); the latter could be differentiated into 24 patients with gNEN-3 (i.e. NET) and 25 gNEN-4 (i.e. NEC) according to Klöppel *et al*. as shown in Table 1 (Klöppel *et al.* 2007). The mean age at ID differed significantly between the patients with gNEN-1 and sporadic gNEN, but in detailed analysis only patients with gNEN-1 were younger compared to gNEN-4 but not to gNEN-3 (Table 2). gNEN revealed a male-to-female ratio (m/f) of 1:1.22 with women constituting the majority of the gNEN-1 (m/f-ratio of 1:2.07) and men the majority of sporadic gNEN (m/f-ratio of 2.43:1).

**Table 1:** Cohort characteristics.

	gNEN-1, *n* (%)	gNEN-2, *n* (%)	gNEN-3, *n* (%)	gNEN-4, *n* (%)	Overall, *n* (%)
Type	86 (60.6)	7 (4.9)	24 (16.9)	25 (17.6)	142 (100)
Associated clinical condition	CAG	G/ZES/MEN-1	Sporadic		–
Grading (WHO 2010)^a^	76	7	20	25	128
G1	59 (77.6)	4 (57.1)	4 (20)	0	67 (52.3)
G2	17 (22.4)	3 (42.9)	16 (80)	1 (4)	37 (28.9)
G3	0	0	0	24 (96)	24 (18.8)
Grade n.k.	10	0	4	0	14
Staging (UICC 2017), clinical	81	n.a.	24	25	130
Stage I	72 (88.9)	n.a.	5 (20.8)	0	77 (59.2)
Stage II	6 (7.4)	n.a.	5 (20.8)	3 (12)	14 (10.8)
Stage III	1 (1.2)	n.a.	4 (16.7)	6 (24)	11 (8.5)
Stage IV	2 (2.5)	n.a.	10 (41.7)	16 (64)	28 (21.5)
Stage n.k.	5	n.a.	0	0	5
Synaptophysin (IHC)	60	3	19	22	104
Positive	59 (98.3)	3 (100)	19 (100)	21 (95.5)	102 (98)
Chromogranin A (IHC)	71	3	21	22	117
Positive	71 (100)	3 (100)	19 (90.5)	16 (72.7)	109 (93.2)
Gastrin (serum)	51	6	9	7	73
Normal	7 (13.7)	0	7 (77.8)	6 (85.8)	20 (27.4)
Elevated	44 (86.3)	6 (100)	2 (22.2)	1 (14.2)	53 (72.6)
n.k.	35	1	15	17	68
Chromogranin A (serum)	52	5	12	15	84
Normal	7 (13.5)	0	2 (16.7)	5 (33.3)	14 (16.7)
Elevated	45 (86.5)	5 (100)	10 (83.3)	10 (66.7)	70 (83.3)
n.k.	34	2	12	9	57
Metastases at ID	86	n.a.	24	24	134
Yes	3 (3.5)	n.a.	12 (50)	18 (75)	33 (24.6)
No	83 (96.5)	n.a.	12 (50)	6 (25)	101 (75.4)
n.k.	0	n.a.	0	1	1
Deaths	69	6	24	23	122
Yes	7 (10.1)	1 (16.7)	11 (45.8)	18 (70.8)	37 (30.3)
No	62 (89.9)	5 (83.3)	13 (54.1)	5 (20.8)	85 (69.7)
n.k.	17	1	0	2	20
Cause of death	6	1	6	13	26
gNEN-related	1 (16.7)	1 (100)	6 (100)	12 (92.3)	20 (70)
Other	5 (83.3)	0	0	1 (7.7)	6 (30)
n.k.	1	0	5	4	10

G1 Ki-67-index ≤2%; G2 Ki-67-index 3-20%; G3 Ki-67-index >20%; Grade n.k. Ki-67-index not available.

^a^Clinical and first available Ki-67-labelling index-based grading; initial and first available information for clinical staging included, staging for patients with gNEN-2 was not applicable due to missing data and non-gastric primary; *n*, amount, % calculated on known data.

CAG, chronic atrophic gastritis; G, gastrinoma; gNEN, gastric neuroendocrine neoplasia; ID, initial diagnosis; IHC, immunhistochemistry; MEN-1, multiple endocrine neoplasia type 1; n.a., not available; n.k., not known; ZES, Zöllinger–Ellison syndrome.

**Table 2:** Clinicopathological characteristics.

	gNEN-1	gNEN-2	gNEN-3	gNEN-4	*P* value^a^		
	Mean ± s.d. (s.e.m.), *n*	Mean ± s.d. (s.e.m.), *n*	Mean ± s.d. (s.e.m.), *n*	Mean ± s.d. (s.e.m.), *n*	gNEN-1 vs -3	gNEN-1 vs -4	gNEN-3 vs -4
Overall, *n*	86	7	24	25	*n.a.*		
Age, years	55.78 ± 13.68 (1.48), 85	41.42 ± 18.98 (7.17), 7	59,55 ± 11.9 (2.43), 24	63.70 ± 11.31 (2.26), 25	0.223	**0.005**	0.21
BMI (kg/m^2^)	26.25 ± 5.21 (0.68), 58	25.57 ± 4.22 (1.88), 5	26.10 ± 4.34 (1.08), 16	26.25 ± 5.21 (1.57), 16	0.920	0.785	0.770
Number of tumours	3.78 ± 4.94 (0.693), 51	14.40 ± 10.21 (4.56), 5	1.29 ± 0.756 (0.28), 7	1.56 ± 1.67 (0.55),9	0.191	0.189	0.698
Size, clinical (mm)	11.00 ± 9.39 (1.26), 55	4.9 ± 5.74 (2.57), 5	19.6 ± 22.90 (10.24), 5	50.71 ± 43.44 (16.42), 7	0.450	**0.052**	0.178
Size, pathological (mm)	8.82 ± 9.52 (1.23), 59	10.40 ± 7.60 (3.40), 5	30.93 ± 36.82 (9.50),15	60.35 ± ,35.32 (8.57), 17	**0.036**	**<0.001**	**0.028**
Ki-67 (%) at ID	2.44 ± 2.58 (0.32), 65	3.9 ± 3.55 (1.45), 6	8.72 ± 7.05 (1.66), 18	58.47 ± 28.34 (5.91), 23	**0.002**	**<0.001**	**<0.001**
Ki-67 (%)^b^	2.39 ± 2.50 (0.29), 73	n.a.	9.85 ± 7.34 (1.64), 20	60.41 ± 26.78 (5.46), 24	**<0.001**	**<0.001**	**<0.001**
Gastrin (µU/mL)^c^	910.02 ± 618.8 (97.84), 40	1824 ± 1935.88 (790.32), 6	101, 35 ± 46.98 (17.75), 7	232.93 ±274.95 (112.24), 6	<**0.001**	**0.012**	0.297
Chromogranin A (µg/L)^c^	1548.76 ± 8138.66 (1213.24), 45	2244.2 ± 2436.06 (1089.44), 5	3212.69 ± 6094.05 (1927.11), 10	2965.96 ± 5344.59 (1379.96), 15	0.546	0.532	0.916

gNEN gastric neuroendocrine neoplasia according to Klöppel *et al.* (2007).

^a^Statistics: *P* value calculated with *t*-test; considered statistically significant if *P* < 0.05; significances given in bold; ^b^Ki-67-labelling index first available values included; ^c^Reference values: serum gastrin (28–185 µU/mL), serum CgA (<102.0 µg/L).

ID, initial diagnosis; Ki-67, Ki-67-labelling index; n.a., not available; sd, standard deviation; s.e.m., standard error of the mean.

### Clinicopathological and treatment characteristics

**Figure 1:**
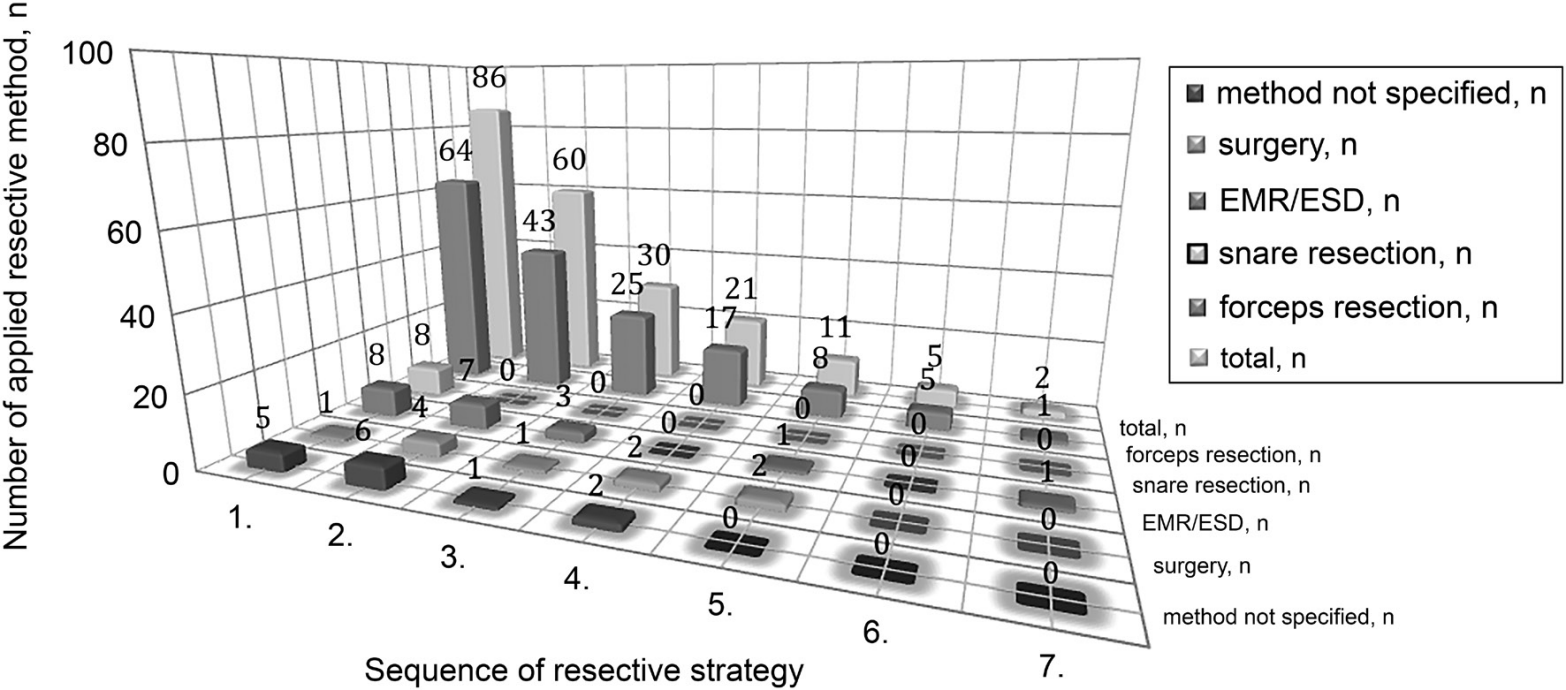
Sequences of resection strategy in patients with gNEN-1. Sequences of resection strategy in patients with gNEN-1. EMR, endoscopic mucosa resection; ESD, endoscopic submucosa dissection.

The variety of applied treatment strategies across all gNEN is shown in Table 3. To further characterize the largest subgroup, the clinicopathological characteristics of patients with gNEN-1, in particular locations, number and size of polyps, associated histology, grading (WHO 2010), pTNM stage and clinical stage (UICC 2017) are provided in reference to resective approach (Table 4) since only very few received medical treatment (SSA) (Table 3). Patients with gNEN-1 received multiple resections during follow-up. Resection by forceps and snare were the predominantly used techniques (*n* = 77; 88.4%), followed by EMR and ESD (*n* = 20; 23.3%). Surgery was an exception in gNEN-1 (*n* = 10; 11.6%), as shown in Fig. 1.

**Table 3:** Therapeutic strategies.

gNEN	ER^a^	SR	SSA	CTx	PRRT
gNEN-1, *n*	154	13	3	0	1
gNEN-3, *n*	9	21	5	8	15
gNEN-4, *n*	9	21	4	30	0
Total, *n*	172	55	12	38	16

gNEN gastric neuroendocrine neoplasia according to Klöppel *et al.* (2007).

^a^Column includes diagnostic interventions without resective intention in gNEN-3 and gNEN-4.

CTx, chemotherapy; EMR, endoscopic mucosa resection; ER, endoscopic resection (including EMR and ESD); ESD, endoscopic submucosa dissection; gNEN, gastric neuroendocrine neoplasia; *n*, number, (all interventions summarized, cumulative figures); PRRT, peptide receptor radionuclide therapy; SR, surgical resection; SSA, somatostatin analogues.

**Table 4:** Clinicopathological characteristics of patients with gNEN-1 according to resective treatment strategy.

Resective treatment strategy	ER, *n* (%)		SR, *n* (%)	Overall, *n* (%)
	ER (any)	EMR/ESD		
gNEN-1, *n* (%)	76 (88.4)	20 (23.3)	10 (11.6)	86 (100)
Sex				
Female	53	12	5	58 (67.4)
Male	23	8	5	28 (32.6)
Endoscopic findings				
Gastric locations of polyps	69	19	11	80 (100)
Corpus	57	14	10	67 (83.75)
Fundus	11	4	1	12 (15)
Cardia	1	1	0	1 (1.25)
Number of polyps	66	17	9	75 (100)
Singular	20	5	5	25 (33.3)
Multiple	46	12	4	50 (66.7)
<5	32	9	6	38 (74.5)
>5	13	3	0	13 (25.5)
Size of polyps	46	14	9	55 (100)
<2 cm	42	12	4	46 (83.6)
>2 cm	4	2	5	9 (16.4)
Grading (WHO 2010)	64	19	7	71 (100)
G1	51	15	5	56 (78.9)
G2	13	4	2	15 (21.1)
G3	0	0	0	0
Grad n.k.	12	1	3	15
Staging (UICC 2017), clinical^a^	68	19	8	76 (100)
Stage I	58	11	3	61 (80.3)
Stage II	9	8	3	12 (15.8)
Stage III	0	0	1	1 (1.3)
Stage IV	1	0	1	2 (2.6)
Stage n.k.	8	1	2	10

G1 Ki-67-index ≤2%; G2 Ki-67-index 3-20%; G3 Ki-67-index >20% according to Grading WHO 2010; specifications in % are given in reference to available information.

^a^At initial diagnosis.

EMR, endoscopic mucosa resection; ER, endoscopic resection (including snare resection and EMR/ESD); ESD, endoscopic submucosa dissection; n.k., not known; SR, surgical resection.

**Figure 2:**
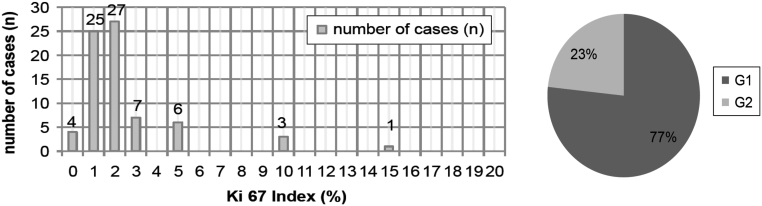
Ki-67-index in patients with gNEN-1. First available Ki-67 index in patients with gNEN-1 according to the WHO grading system 2010 (Bosman *et al.* 2010): G1 Ki-67 <3%; G2 >3–20%; G3 >20%.

Grading based on Ki-67-labelling index (first-available) was recorded in 128 (90.1%) gNEN (Table 1). In total, 67 patients had G1 (52.3%), 37 had G2 (28.9%) and 24 presented with G3 (18.8%). Ki67-index was available in 73 of 86 gNEN-1 (84.8%). In this subgroup 77% of all patients had a G1, and 23% had a G2 NET, while no grade 3 NEN was reported (Fig. 2). Sufficient clinical information for adequate clinical staging was available in 81 of 86 with gNEN-1 (94%) and in all patients with sporadic gNEN (Table 1). At initial diagnosis (ID) clinical staging according to UICC 2017 was available in 130 of 142 patients (91.5%). In total the majority presented with stage I (59.2%), stage II and III (19.3%) and stage IV (21.5%). In 76 (88.4%) of all patients with gNEN-1 information for staging was given (Table 4). Besides age (*P* = 0.035) and amount of polyps (*P* = 0.001) mainly tumour size (macroscopic, *P* = 0.044, and microscopic, *P* = 0.014) determined the therapeutic strategy in gNEN-1 (Supplementary Table 1, see section on supplementary data given at the end of this article).

Staining data were available in 104 of 141 (73.7%) patients for synaptophysin and 117 of 141 (82.9%) regarding CgA. Serum gastrin levels were available in 73 of 141 patients (51.7%) and in 84 of 141 patients (59.6%) CgA-levels were given (Table 1). Serum gastrin levels were significantly higher in gNEN-1 than in gNEN-3 and gNEN-4 (*P* < 0.001 and *P* = 0.012), while CgA levels not (*P* = 0.546 and *P* = 0.532; Table 2). However, serum gastrin levels were not statistically significant neither for metastatic and non-metastatic, low- or intermediate grade (G1 vs G2) nor treatment (ER vs SR) in gNEN-1 (*P* = 0.777, *P* = 0.049, *P* = 0.749).

In total, 36 of 142 patients (25.4%) died during follow-up. In 20 of 26 cases (77%) with known cause of death, death was NEN related. Seven patients with gNEN-1 (8%) died during follow-up, but only one death was NEN related (liver failure due to extensive hepatic metastasis, 1.2%).

### Survival analysis

**Figure 3:**
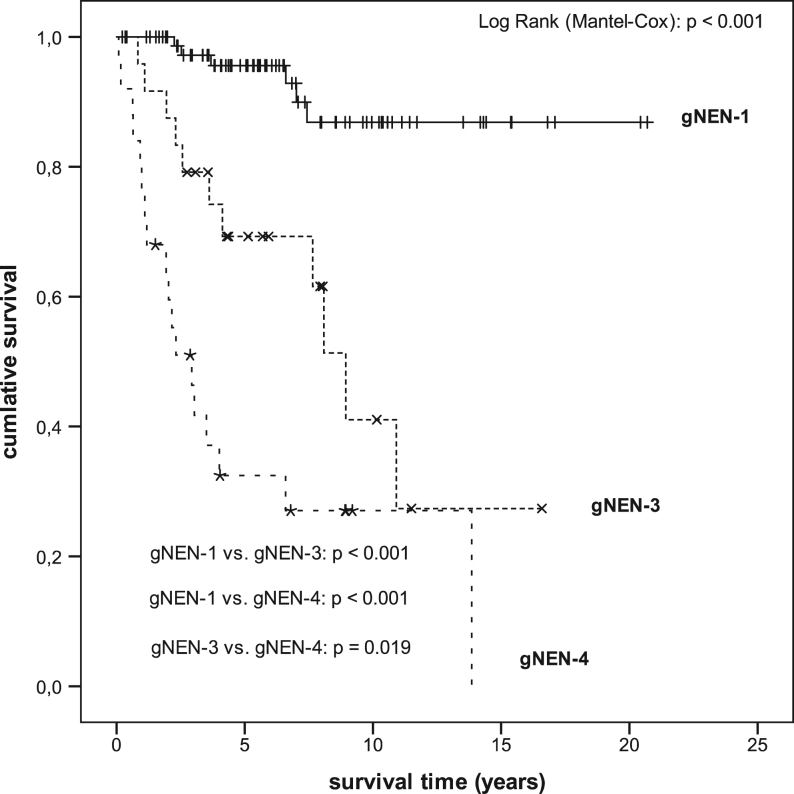
Kaplan–Meier analysis of overall survival of patients with gNEN according to the refined typification (Klöppel *et al.* 2007). Kaplan–Meier analysis of overall survival (with endpoint any death cause) of patients with gNEN according to the classification proposed by Klöppel *et al.* (2007) (gNEN-1 vs gNEN-3 vs gNEN-4). s.e.m., standard error of mean; CI, confidence interval; *one death reported without available survival time. Mean survival (years); SEM (CI 95%): gNEN-1: 18.713; 0.777 (17.195–20.241); gNEN-3: 9.118; 1.401 (6.372–11.863); gNEN-4: 5.316; 11.164 (3.035–7.596); overall: 14.216; 0.935 (12.383–16.049).

**Figure 4:**
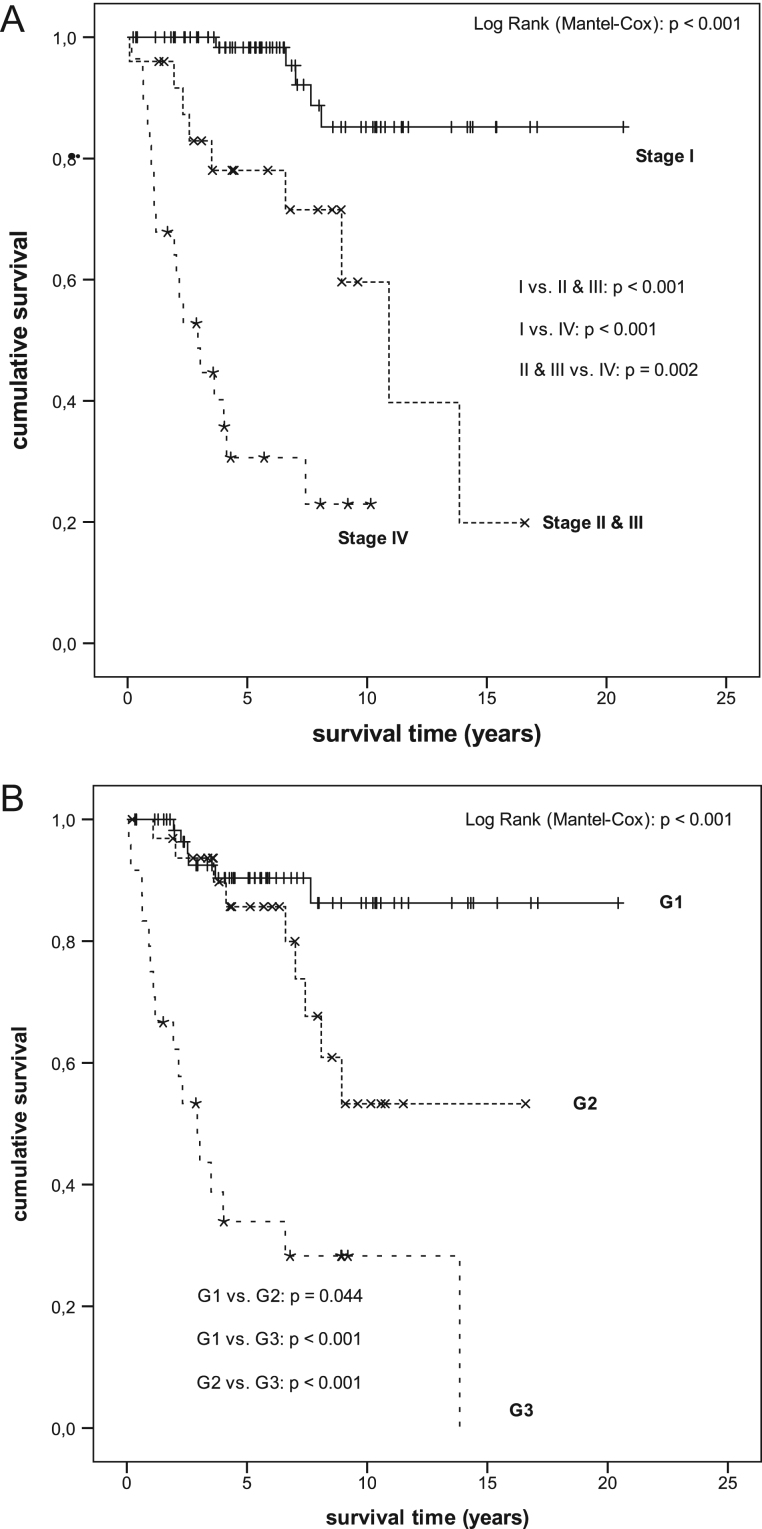
Kaplan–Meier analysis of overall survival of patients with gNEN according to the UICC 2017 staging and WHO 2010 grading system. (A) Kaplan–Meier analysis of overall survival (with endpoint any death cause) of patients with gNEN depending on the regrouped UICC 2017 staging system, i.e. due to few cases patients with stages II and III were regrouped into stages II and III. s.e.m., standard error of mean; CI, confidence interval; *one death reported without available survival time. Mean survival (years); s,e,m, (CI 95%): Stage I: 18.633; 0.855 (16.987–20.338); stages II and III: 10.183; 1.371 (7.495–12.871); Stage IV: 4.283; 0.737 (2.839–5.727); overall: 14.297; 0.962 (12.412–16.182). (B) Kaplan–Meier analysis of overall survival of patients with gNEN according to the WHO 2010 grading system (Bosman *et al.* 2010). Kaplan–Meier analysis of overall survival (with endpoint any death cause) of patients with gNEN according to the WHO 2010 grading system, i.e. clinical and first available Ki-67-index based grading; G1 Ki-67-index ≤2%; G2 Ki-67-index 3-20%; G3 Ki-67-index >20%. s.e.m., standard error of mean; CI, confidence interval; *one death reported without available survival time. Mean survival (years); s.e.m. (CI 95%): G1: 18.194; 0.873 (16.484–19.904); G2: 11.740; 1.240 (9.310–14.141); G3: 5.468; 1.207 (3.102–7.833); overall: 14.105; 0.954 (12.235–15.975).

Total mean OS (any cause of death) for all patients was 14.216 years (s.e.m. 0.935; CI 12.283–16.049). Mean OS differed statistically significant between all gNEN-types as presented in Fig. 3 (gNEN-1 vs gNEN-3: *P* < 0.001; gNEN-1 vs gNEN-4: *P* < 0.001; gNEN-3 vs gNEN-4: *P* = 0.019). In 128 patients of 142 (90.1%), the updated UICC 2017 tumour staging system was available (stage I: *n* = 75, stage II/III: *n* = 25 and stage IV: *n* = 28); due to few numbers stages II and III were analysed combined for OS as shown in Fig. 4A. Mean OS differed statistically significant between all stages (I vs III: *P* = 0.001; I vs IV: *P* < 0.001; II/III vs IV: *P* = 0.002).

Outcome according to maximum Ki-67-labelling index-based grading of the WHO (2010) grading system was analysed in 119 of 142 patients (83.8%). Mean OS differed statistically significant between all grades (G1 vs G2: *P* = 0.044; G1 vs G3: *P* < 0.001; G2 vs G3: *P* < 0.001; Fig. 4B).

**Figure 5:**
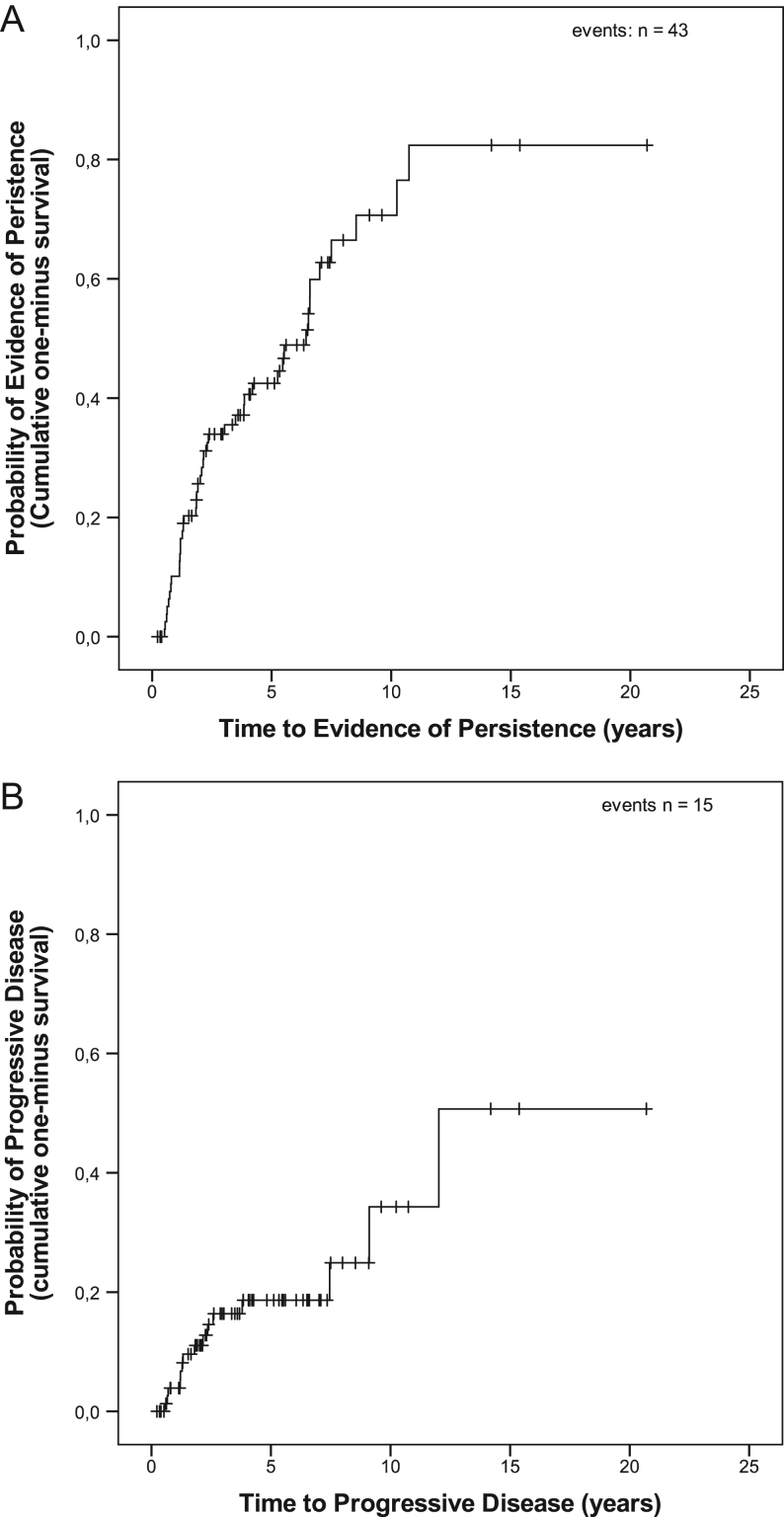
Kaplan–Meier analysis of secondary endpoints. (A) Time to evidence of persistence (TTEP) of patients with gNEN-1; EP, Evidence of Persistence defined as endoscopic and histopathologic rediscovery of gNEN-1 polyps during follow-up. s.e.m., standard error of mean; CI, confidence interval. Mean TTEP (years); s.e.m. (CI 95%): gNEN-1: 7.56; 1.098 (5.416–9.72). (B) Time to progressive disease (TTPD) in patients with gNEN-1; PD, progressive disease defined as transformation of histopathological grade (WHO 2010) and/or increased clinicopathological staging (pTNM/cTNM) during follow-up. s.e.m., standard error of mean; CI, confidence interval. Mean TTPD (years); SEM (CI 95%): gNEN-1:13.83; 1.75 (10.38–17.28).

Because outcome appeared to be almost unlimited for gNEN-1 alternative endpoints other than OS for characterizing long-term outcome were studied. In 43 of 82 patients with gNEN-1 (52.4%) evidence of persistence (EP) was confirmed by endoscopic and histopathological follow-up; calculated mean TTEP was 7.56 years (Fig. 5A). In addition, for only 15 of 82 patients (18.3%), a disease progression (PD) was documented and a mean TTPD of 13.83 years was calculated (Fig. 5B).

### Cox regression analysis of the whole cohort for survival

The typification of gNEN according to Klöppel *et al*. was shown to be a strong independent predictor of outcome for both overall as well as NEN-related survival with increased hazard ratios for death depending on typing and grading (Table 5). Grading proved to be an independent predictor of outcome with a significantly increased risk for overall death in G3 and for NEN-related survival in G2 as well as G3 compared to NET-G1 as well. Similarly, the UICC 2017 staging system demonstrated an increased risk of death for advanced stages (Table 5).

**Table 5:** Cox regression analysis of overall and gNEN-related survival in all patients with gNEN and TTEP and TTPD of patients with gNEN-1.

Risk stratifiers	Hazard ratio (CI 95%)	*P* value
Overall survival		
Typing		
gNEN-1^a^		
gNEN-3	**4.776** (1.720–13.257)	**0.003**
gNEN-4	**11.139** (4.076–30.439)	**<0.001**
Grading (WHO 2010), at ID		
G1^a^		
G2	1.764 (0.635–4.897)	0.276
G3	**4.881** (1.866–12.769)	**0.001**
Grading (WHO 2010)^b^		
G1^a^		
G2	2.328 (0821–6.601)	0.112
G3	**7.417** (2.757–19.951)	**<0.001**
Staging (UICC 2017–regrouped)		
Stage I^a^		
Stage II and III	**4.447** (1.455–13.592)	**0.009**
Stage IV	**12.957** (4.489–37.399)	**<0.001**
NEN-related survival		
Typing		
gNEN-1^a^		
gNEN-3	**18.335** (2.116–155.164)	**0.008**
gNEN-4	**67.203** (8.013–563.632)	**<0.001**
Number of polyps		
Singular^a^ vs multiple	**0.118 (**0.014–0.984**)**	**0.048**
Size		
<2 cm^a^ vs >2 cm	6.202 (0.968–39.746)	0.054
Ki-67-labelling index	**1.017** (1.003–1.032)	**0.02**
Grading (WHO 2010), at ID		
G1^a^		
G2	**9.404** (1.121–78.890)	**0.039**
G3	**25.203** (3.044–208.664)	**0.003**
Grading (WHO 2010)^b^		
G1^a^		
G2	**9.459** (1.129–79.248)	**0.038**
G3	**37.728** (4.613–208.599)	**0.001**
TTEP		
Age, at ID	0.987 (0.962–1.011)	0.288
Gender		
Female^a^ vs male	1.43 (0.747–2.738)	0.281
Number of polyps		
Singular^a^ vs multiple	**2.524** (1.191–5.349)	**0.016**
Number of polyps		
<5^a^ vs >5	**3.725** (1.716–8.089)	**0.001**
TTPD		
Age, at ID	0.973 (0.931–1.018)	0.234
Gender		
Female^a^ vs male	2.628 (0.925–7.47)	0.07
Number of polyps		
Singular^a^ vs multiple	1.387 (0.414–4.646)	0.596
<5^a^ vs. >5	**3.541** (1.031–12.161)	**0.045**

gNEN gastric neuroendocrine neoplasia according to Klöppel *et al.* (2007); G1 Ki-67-index ≤2%; G2 Ki-67-index 3-20%; G3 Ki-67-index >20% according to grading system of WHO 2010.

^b^Grading with first available Ki-67-labelling index; staging according to UICC 2017; TTEP, time to evidence of persistence; TTPD, time to progressive disease; ^a^reference variable; CI confidence interval; *P* value <0.05 considered statistically significant; significances and related hazards presented in bold, an age- and gender-adjusted model was applied.

### Cox regression analysis of gNEN-1 for secondary endpoints

The multivariate analysis (age- and gender-adjusted model) showed a higher hazard of EP if polyps were multiple (*P* = 0.016) or in case of more than five polyps (*P* = 0.001). The amount of more than five polyps at ID was associated with a 3.4-fold increased risk (*P* = 0.045) for PD underlining the chronic condition of CAG and the necessity of endoscopic surveillance.

## Discussion

This single-centre study presents a large clinical cohort of gNEN and describes the three most important independent prognostic parameters: pathophysiological typification including highly malignant grade 3 (gNEC, gNEN-4 type), WHO grading according to Ki67-proliferative index and UICC staging. Furthermore, on detailed analysis of the mostly indolent gNEN-1 subgroup (i.e. so-called ECLomas), we demonstrate both, excellent long-term outcome but also the slowly progressive nature due to the underlying condition of CAG ultimately leading to disease persistence as well as progression and even metastasis and death. Therefore, these results are a strong indicator that lifelong follow-up is warranted as recommended by current guidelines and that long-term outcome of gNEN-1 is excellent if appropriate classification and treatment stratification are applied.

In our clinical cohort, the recorded baseline data were comparable to figures reported in the literature (La Rosa *et al.* 2011, Saund *et al.* 2011, Thomas *et al.* 2013, Kim *et al.* 2016, Liang *et al.* 2016, Shen *et al.* 2016, Chung *et al.* 2018, Vanoli *et al.* 2018, Yang *et al.* 2018). Our analysis demonstrated that the pathophysiological typification initially proposed by Klöppel *et al*., the UICC 2017 TNM staging and the WHO 2010 grading classifications are of great prognostic relevance in gNEN (Klöppel *et al.* 2007, Bosman *et al.* 2010, Delle Fave *et al.* 2016, Gospodarowicz *et al.* 2017). Of note, the vast majority of gNEN with elevated serum gastrin levels were in fact gNEN-1 (Tables 1 and 2) significantly associated with CAG and thus also associated with better outcome figures (see below). However, analyses did not show that elevated gastrin levels increased the risk for progressive disease in gNEN-1, while the amount of polyps did by 3.5-fold (Table 5). The pathophysiological typification according to Klöppel *et al*. showed a strong significant difference in OS of all types (Fig. 3). In addition, patients with gNEN-3 as compared to gNEN-4 can be stratified according to their considerably lower risk of death (Table 5).

Our data show that the Ki-67-labelling index is correlated reciprocally with prognosis in gNEN like it is for upper GEP-NEN (Pape *et al.* 2008*b*). The majority of gNEN-1 had G1; G2 grades were on the lower edge of the scale (Fig. 2), while no G3 was found, alike to others (Campana *et al.* 2016). The Ki67 index-based grading showed a statistically significant difference in OS between all grades (Fig. 4B and Table 5A, B).

Further analysis revealed that UICC 2017 staging statistically separated stage IV from all other stages and indicated an increased relative risk of death. The combination of locally advanced and locoregionally progressed stages II and III showed likewise compared to stage I and stage IV a statistically different OS, that is a 13-fold risk of death in patients with stage IV disease (Fig. 4A and Table 5). These results are supported by prior findings implicating that size, invasiveness and disease spread have a significant impact on a poorer outcome (Rindi *et al.* 1999, Pape *et al.* 2008*b*, Saund *et al.* 2011). In our cohort statistically significant correlations between amount, size, Ki67 index and type were given (Table 2), but the relevance of the amount of infiltrated lymph nodes due to lack of specific data could not be evaluated; however, the predictive value for survival was shown recently (Pak *et al.* 2019). The major therapeutic approach in all gNEN was resection or systemic in advanced irresectable gNEN (Table 3). In gNEN-1, local resection was predominantly performed, while surgical resections as well as palliative approaches were more often conducted in sporadic gNEN (Fig. 1 and Tables 3, 4). In contrast, patients with sporadic gNEN had relatively advanced stages (61.3%: 30/49) which explains the high rate of surgery as well as chemotherapy in this subgroup, because only in gNEN-3 with low risk profile ESD might be a safe option (Kwon *et al.* 2013). Regarding the treatment and survival of gNEN-4 (i.e. sporadic gNEC) our results are comparable to the literature on gNEC and GEP-NEC in general (Ishida *et al.* 2013, Xu *et al.* 2014, Heetfeld *et al.* 2015, Tang *et al.* 2015, Liu *et al.* 2017). The type dependent 5- and 10-year OS rates of our cohort were as follows: gNEN-1 95.6% and 86.9%, in gNEN-3 69.3.8% and 41.0% and in gNEN-4 33.8% and 28.2% respectively.

The reported 5-year OS rates of gNEC are heterogenous with 35–48% (Ishida *et al.* 2013, Xu *et al.* 2014, Tang *et al.* 2015). Unfortunately heuristic testing of various alternative Ki67-thresholds showed no benefits for stratification of patients at low- and mid-risk, but a 2.38-fold increased risk of death in gNEC with Ki67 index >57.5% was recently reported (Xie *et al.* 2017). Hence, especially grading determines prognosis in gNEN supporting a type differentiation of gNEN-3 (gNET) from gNEN-4 (gNEC).

In gNEN-1 the evaluation of recurrence and progression remains difficult because of several methodological problems such as multiplicity, incomplete resection by forceps, observer bias and last but not least chronic proliferative stimulation in CAG by hypergastrinemia (Merola *et al.* 2012). However, the excellent OS of gNEN-1 (Fig. 3) is comparable with cohorts treated more aggressively (Borch *et al.* 2005, Gladdy *et al.* 2009). Thus, our findings support the trend towards a more cautious approach in low-risk gNEN-1 (<1 cm, <5 lesions, G1–G2) with ER and annual surveillance (Ruszniewski *et al.* 2006, Delle Fave *et al.* 2016).

Otherwise our findings refuse any oversimplification of gNEN-1 (Rappel *et al.* 1995, Ravizza *et al.* 2007). At least a concise diagnostic work- and follow-up is warranted to identify the individual risk profile (Ichikawa *et al.* 2003, Ruszniewski *et al.* 2006, Chen *et al.* 2012, Kim *et al.* 2014). Our data state a 3.4-fold increased risk of progression in patients with more than five gNEN-1 lesions, but probably due to stringent surveillance without consequences for long-term survival.

Surgery is controversially discussed regarding gNEN-1. Multiplicity and recurrence after resection have been assumed to justify even antrectomy in gNEN-1 in the past (Gladdy *et al.* 2009). However, in our gNEN-1 cohort surgery was only applied in 11.6% with no deleterious results in long-term survival suggesting a less invasive strategy to be sufficient in most gNEN-1. Independent from gNEN type a gastrectomy has been recommended in large (>2 cm), deeply infiltrating G2 tumours and widespread lesions or in the case of recurrence despite prior ER, EMR/ESD or local SR, and in some cases of metastasis (Ruszniewski *et al.* 2006, Kaltsas *et al.* 2014, Delle Fave *et al.* 2016). Our data support this more invasive approach due to a six-fold increased risk for NEN-related death in gNEN lesions bigger than 2 cm (Table 5).

Reports on metastasis rates in gNEN-1 are heterogenous: ranging from 4.8 to 19.2% (Sagatun *et al.* 2016, Vanoli *et al.* 2018). In our cohort in six cases (7%) metastatic disease was detected in gNEN-1 (50% of such during follow-up, see Supplementary Table 2), while only one NEN-related death occurred. Both clinical constellations are extremely rare (Grozinsky-Glasberg *et al.* 2013). Both alternative endpoints for gNEN-1 indicate the chronic and potentially progressive clinical course of gNEN-1 requiring a long-term follow-up: in 50.7% PD occurred after a mean of 12 years but without consequence for disease-specific survival (Fig. 5). The persistence rate indicates the clinical chronicity of gNEN-1 with 44.5% at 5, 76.5% at 10 and 82.4% at 15 years of follow-up in our cohort (Fig. 5A), similarly documented with a 63.6% mean recurrence rate (RR) after 8 months and a recurrence free survival of 24 months or even a RR of 18% in gNEN-1 (Merola *et al.* 2012, Uygun *et al.* 2014). These data demonstrate the possibility of excellent OS of gNEN-1 treated by local resection and endoscopic follow-up. Thus, surgery may only be justifiable in the very rare patients with advanced or even metastatic gNEN-1 (Kaltsas *et al.* 2014, Delle Fave *et al.* 2016).

Somatostatin analogues (SSAs) have not been used routinely in our cohort of gNEN-1 mainly due to lack of evidence for prolongation of lifetime, relatively high costs and thus an unclear cost-benefit ratio in gNEN-1. However, long-term outcome was extremely favourable for gNEN-1 justifying the watch-and-wait approach (Kulke *et al.* 2010) with mostly endoscopic management in contrary to antrectomy (Borch *et al.* 2005, Guillem 2005, Dakin *et al.* 2006) or systemic antiproliferative SSA treatments (Grozinsky-Glasberg *et al.* 2008, Thomas *et al.* 2013, Massironi *et al.* 2015, Campana *et al.* 2016). However, in rare metastatic gNEN-1 with low Ki-67-labelling index and proven SSTR2 expression (Delle Fave *et al.* 2016), SSA can be considered as palliative option for symptom and growth control (Table 3). Promising alternatives for disease control might be Gastrin/CCK2 Receptor antagonists (Netazepide) or mTOR inhibitors (Lohneis *et al.* 2014, Boyce & Thomsen 2015). So far, the presented data constrain that repeated local resections by short and mid-term endoscopic follow-up is a pragmatic and cost-effective approach which leads to a very good clinical outcome of gNEN-1, even in cases of recurrence or progression due to ongoing HG caused by CAG. However, follow-up intervals are part of ongoing controversy, probably because of inconsistent reports on the adenocancerogenic potential of CAG and intestinal metaplasia itself (Chen *et al.* 2015, Lahner *et al.* 2015, Xie *et al.* 2017). Neither adenocarcinoma nor a life-threatening progression of gNEN-1 was documented during follow-up in our cohort. Thus, stringent surveillance is mandatory in gNEN-1 due to the risk of progressive disease and metastases, which while unlikely to occur, have grave consequences if missed.

### Limitations

The results of our study may not be generalized due to some limitations such as immanent selection bias of retrospective analysis as well as specific definitions or alternative endpoints, which will limit the degree of comparability with other studies. Furthermore, a referral bias to our ENETS Centre of Excellence leading to more advanced patients with a poorer prognosis can be assumed (Pape *et al.* 2008*a*). In addition, limited histopathological information was rated as a random limitation due to histopathological diagnoses made in external institutions. Tumour tissue for additional Ki-67-labelling was unavailable because of biopsy specimen or lack of availability from the allocating institution. Moreover, the treatment with forceps often implicates a resection effort but not necessarily the R0-result in the context of gNEN-1 (because of multiplicity or stealth submucosal invasion), challenging the oncologic concept of recurrence in this kind of neoplasia.

### Conclusion

In summary, our data demonstrate that the typification proposed by Klöppel *et al.* (2007), the new UICC (2017) classification system for histopathological and clinical staging and the WHO grading system (2010) are valid for risk stratification of gNEN in routine patient management. While the course of gNEN-1 is mostly benign with rare low risk of metastasis and death, the prognosis of sporadic gNEN can be successfully stratified by refined Ki67-labelling index-based typing.

## Supplementary Material

Supplemental Tables 1. Clinicopathologic characteristics of gNEN-1 according to resective treatment strategy Legend: ER endoscopic resection, EMR endoscopic mucosa resction, ESD endoscopic submucosa dissection, SR surgery, SD standard deviation, SEM standard error of mean, n number, BMI body mass index, macro macroscopic, micro microscopic, * significances in bold, considered statistically significant if p < 0.05 Reference Values: Gastrin µU/ml (28-185), CgA µg/l (<102.0)

Supplemental Table 2. Clinicopathologic Characteristics of patients with metastatic gNEN- 1 Legend: f female, m male, Age in years, ID initial diagnosis, FU follow up, H hepatic, LN lymphnode, EUS endoscopic ultrasound, SSR Somatostatin receptor, SSA Somatostatin Analoga, BII Billroth II, ER Endoscopic resection, S surgery, PRRT Peptide receptor radionuclid therapy, Ki-67 index, (*) maximal available

## Declaration of interest

The authors declare that there is no conflict of interest that could be perceived as prejudicing the impartiality of the research reported.

## Funding

The study was supported financially neither by grant nor by any company.

## Author contribution statement

Stephan Felder: data maintenance and analysis, methodology, investigation, formal analysis and writing of the first draft, writing and editing the paper. Henning Jann: methodology, validation of data, review, writing the paper, supervision. Ruza Arsenic: validation of tumour tissue and immunochemistry, review. Tim Denecke: validation of imaging results, review. Vikas Prasad: validation of nuclear imaging results, review. Barbora Knappe-Drzikova: methodology, validation of data, review. Sebastian Maasberg: validation of data, review. Andreas Pascher: validation of data, review. Bertram Wiedenmann: review, resources, supervision. Marianne Pavel: review, resources, supervision. Ulrich-Frank Pape: conceptualisation, methodology, validation of data, formal analysis, review, writing the paper, resources, supervision.

## References

[bib1] Askanazy M 1923 Zur Pathogenese der Magenkrebse und über ihren gelegentlichen Ursprung aus angeborenen epithelialen Keimen in der Magenwand (Schluß aus Nr. 1.). Deutsche Medizinische Wochenschrift 49 . (10.1055/s-0028-1131778)

[bib2] Borch K Ahren B Ahlman H Falkmer S Granerus G Grimelius L 2005 Gastric carcinoids: biologic behavior and prognosis after differentiated treatment in relation to type. Annals of Surgery 242 . (10.1097/01.sla.0000167862.52309.7d) PMC1357706 15973103

[bib3] Bosman FT Carneiro F Hruban RH Theise ND 2010 WHO Classification of Tumours of the Digestive System. Geneva, Switzerland: World Health Organization.

[bib4] Boyce M Thomsen L 2015 Gastric neuroendocrine tumors: prevalence in Europe, USA, and Japan, and rationale for treatment with a gastrin/CCK2 receptor antagonist. Scandinavian Journal of Gastroenterology 50 . (10.3109/00365521.2015.1009941) 25665655

[bib5] Campana D Ravizza D Ferolla P Faggiano A Grimaldi F Albertelli M Berretti D Castellani D Cacciari G Fazio N, ***et al*** 2016 Clinical management of patients with gastric neuroendocrine neoplasms associated with chronic atrophic gastritis: a retrospective, multicentre study. Endocrine 51 . (10.1007/s12020-015-0584-z) 25814125

[bib6] Campana D Ravizza D Ferolla P Faggiano A Grimaldi F Albertelli M Ricci C Santini D Brighi N Fazio N, ***et al*** 2017 Risk factors of type 1 gastric neuroendocrine neoplasia in patients with chronic atrophic gastritis. A retrospective, multicentre study. Endocrine 56 . (10.1007/s12020-016-1099-y) 27592118

[bib8] Chen WF Zhou PH Li QL Xu MD Yao LQ 2012 Clinical impact of endoscopic submucosal dissection for gastric neuroendocrine tumors: a retrospective study from mainland China. Scientific World Journal 2012 869769 (10.1100/2012/869769) 23326217 PMC3541571

[bib7] Chen WC Warner RR Ward SC Harpaz N Divino CM Itzkowitz SH Kim MK 2015 Management and disease outcome of type I gastric neuroendocrine tumors: the Mount Sinai experience. Digestive Diseases and Sciences 60 . (10.1007/s10620-014-3410-1) 25399327

[bib9] Chung CS Tsai CL Chu YY Chen KC Lin JC Chen BC Sun WC Yen HH Chen CY Wu IC, ***et al*** 2018 Clinical features and outcomes of gastric neuroendocrine tumors after endoscopic diagnosis and treatment: a Digestive Endoscopy Society of Tawian (DEST). Medicine 97 e12101 (10.1097/MD.0000000000012101) 30235663 PMC6160255

[bib10] Dakin GF Warner RR Pomp A Salky B Inabnet WB 2006 Presentation, treatment, and outcome of type 1 gastric carcinoid tumors. Journal of Surgical Oncology 93 . (10.1002/jso.20468) 16550587

[bib11] Dasari A Shen C Halperin D Zhao B Zhou S Xu Y Shih T Yao JC 2017 Trends in the incidence, prevalence, and survival outcomes in patients with neuroendocrine tumors in the United States. JAMA Oncology 3 . (10.1001/jamaoncol.2017.0589) PMC5824320 28448665

[bib12] Delle Fave G O'Toole D Sundin A Taal B Ferolla P Ramage JK Ferone D Ito T Weber W Zheng-Pei Z, ***et al*** 2016 Enets consensus guidelines update for gastroduodenal neuroendocrine neoplasms. Neuroendocrinology 103 . (10.1159/000443168) 26784901

[bib13] Gladdy RA Strong VE Coit D Allen PJ Gerdes H Shia J Klimstra DS Brennan MF Tang LH 2009 Defining surgical indications for type I gastric carcinoid tumor. Annals of Surgical Oncology 16 . (10.1245/s10434-009-0687-y) 19727959

[bib14] Gospodarowicz MK Brierley JD Wittekind C 2017 TNM Classification of Malignant Tumours. Hoboken, NJ, USA: John Wiley & Sons.

[bib15] Grozinsky-Glasberg S Kaltsas G Gur C Gal E Thomas D Fichman S Alexandraki K Barak D Glaser B Shimon I, ***et al*** 2008 Long-acting somatostatin analogues are an effective treatment for type 1 gastric carcinoid tumours. European Journal of Endocrinology 159 . (10.1530/EJE-08-0420) 18662970

[bib16] Grozinsky-Glasberg S Thomas D Strosberg JR Pape UF Felder S Tsolakis AV Alexandraki KI Fraenkel M Saiegh L Reissman P, ***et al*** 2013 Metastatic type 1 gastric carcinoid: a real threat or just a myth? World Journal of Gastroenterology 19 . (10.3748/wjg.v19.i46.8687) PMC3870515 24379587

[bib61] Guillem P 2005 [Gastric carcinoid tumours. Is there a place for antrectomy?]. Annales de Chirurgie 130 . 10.1016/j.anchir.2005.03.010 15890310

[bib17] Heetfeld M Chougnet CN Olsen IH Rinke A Borbath I Crespo G Barriuso J Pavel M O'Toole D Walter T, ***et al*** 2015 Characteristics and treatment of patients with G3 gastroenteropancreatic neuroendocrine neoplasms. Endocrine-Related Cancer 22 . (10.1530/ERC-15-0119) 26113608

[bib18] Ichikawa J Tanabe S Koizumi W Kida Y Imaizumi H Kida M Saigenji K Mitomi H 2003 Endoscopic mucosal resection in the management of gastric carcinoid tumors. Endoscopy 35 . (10.1055/s-2003-37256) 12584637

[bib19] Ishida M Sekine S Fukagawa T Ohashi M Morita S Taniguchi H Katai H Tsuda H Kushima R 2013 Neuroendocrine carcinoma of the stomach: morphologic and immunohistochemical characteristics and prognosis. American Journal of Surgical Pathology 37 . (10.1097/PAS.0b013e31828ff59d) 23759931

[bib21] Kaltsas G Grozinsky-Glasberg S Alexandraki KI Thomas D Tsolakis AV Gross D Grossman AB 2014 Current concepts in the diagnosis and management of type 1 gastric neuroendocrine neoplasms. Clinical Endocrinology 81 . (10.1111/cen.12476) 24750249

[bib23] Kim HH Kim GH Kim JH Choi MG Song GA Kim SE 2014 The efficacy of endoscopic submucosal dissection of type I gastric carcinoid tumors compared with conventional endoscopic mucosal resection. Gastroenterology Research and Practice 2014 253860 (10.1155/2014/253860) 24693280 PMC3947882

[bib22] Kim BS Park YS Yook JH Kim BS 2016 Comparison of the prognostic values of the 2010 WHO classification, AJCC, 7th ed., and ENETS classification of gastric neuroendocrine tumors. Medicine 95 e3977. 27472674 10.1097/MD.0000000000003977 PMC5265811

[bib24] Klimstra DS Modlin IR Adsay NV Chetty R Deshpande V Gonen M Jensen RT Kidd M Kulke MH Lloyd RV, ***et al*** 2010 Pathology reporting of neuroendocrine tumors: application of the Delphic consensus process to the development of a minimum pathology data set. American Journal of Surgical Pathology 34 . (10.1097/PAS.0b013e3181ce1447) 20118772

[bib25] Klöppel G Rindi G Anlauf M Perren A Komminoth P 2007 Site-specific biology and pathology of gastroenteropancreatic neuroendocrine tumors. Virchows Archiv 451 (Supplement 1) S9–S27. (10.1007/s00428-007-0461-0) 17684761

[bib26] Kulke MH Anthony LB Bushnell DL de Herder WW Goldsmith SJ Klimstra DS Marx SJ Pasieka JL Pommier RF Yao JC, ***et al*** 2010 NANETS treatment guidelines: well-differentiated neuroendocrine tumors of the stomach and pancreas. Pancreas 39 . (10.1097/MPA.0b013e3181ebb168) PMC3100728 20664472

[bib27] Kwon YH Jeon SW Kim GH Kim JI Chung IK Jee SR Kim HU Seo GS Baik GH Choi KD, ***et al*** 2013 Long-term follow up of endoscopic resection for type 3 gastric NET. World Journal of Gastroenterology 19 . (10.3748/wjg.v19.i46.8703) PMC3870517 24379589

[bib28] La Rosa S Inzani F Vanoli A Klersy C Dainese L Rindi G Capella C Bordi C Solcia E 2011 Histologic characterization and improved prognostic evaluation of 209 gastric neuroendocrine neoplasms. Human Pathology 42 . (10.1016/j.humpath.2011.01.018) 21531442

[bib29] Lahner E Esposito G Pilozzi E Galli G Corleto VD Di Giulio E Annibale B 2015 Gastric cancer in patients with type I gastric carcinoids. Gastric Cancer 18 . (10.1007/s10120-014-0393-8) 24890255

[bib30] Liang W Gao Y Li J Cui J Xi H Cai A Chen L 2016 Clinicopathologic features and prognostic analysis of 104 patients with gastric neuroendocrine neoplasms. Chinese Journal of Gastrointestinal Surgery 19 . 27112478

[bib31] Liu DJ Fu XL Liu W Zheng LY Zhang JF Huo YM Li J Hua R Liu Q Sun YW 2017 Clinicopathological, treatment, and prognosis study of 43 gastric neuroendocrine carcinomas. World Journal of Gastroenterology 23 . (10.3748/wjg.v23.i3.516) PMC5291857 28210088

[bib39] Lohneis P Griniak K Felder S Pape UF Dietel M & Arsenic R 2014 Immunohistochemical analysis of mTOR pathway expression in gastric neuroendocrine tumors. Journal of Clinical and Experimental Pathology 4 . (10.4172/2161-0681.1000173)

[bib32] Massironi S Sciola V Spampatti MP Peracchi M Conte D 2009 Gastric carcinoids: between underestimation and overtreatment. World Journal of Gastroenterology 15 . (10.3748/wjg.15.2177) PMC2682231 19437556

[bib33] Massironi S Zilli A Fanetti I Ciafardini C Conte D Peracchi M 2015 Intermittent treatment of recurrent type-1 gastric carcinoids with somatostatin analogues in patients with chronic autoimmune atrophic gastritis. Digestive and Liver Disease 47 . (10.1016/j.dld.2015.07.155) 26321479

[bib34] Merola E Sbrozzi-Vanni A Panzuto F D'Ambra G Di Giulio E Pilozzi E Capurso G Lahner E Bordi C Annibale B, ***et al*** 2012 Type I gastric carcinoids: a prospective study on endoscopic management and recurrence rate. Neuroendocrinology 95 . (10.1159/000329043) 21811050

[bib35] Oberndorfer S 1907 Karzinoide tumoren des Dünndarms. Frankfurter Zentralblatt für Pathologie 1 .

[bib36] Pak LM Yang T Wang J 2019 Further classification for node-positive gastric neuroendocrine neoplasms. Journal of Gastrointestinal Surgery 23 . (10.1007/s11605-018-3845-3) 29951901

[bib37] Pape UF Berndt U Muller-Nordhorn J Bohmig M Roll S Koch M Willich SN Wiedenmann B 2008a Prognostic factors of long-term outcome in gastroenteropancreatic neuroendocrine tumours. Endocrine-Related Cancer 15 . (10.1677/ERC-08-0017) 18603570

[bib38] Pape UF Jann H Muller-Nordhorn J Bockelbrink A Berndt U Willich SN Koch M Rocken C Rindi G Wiedenmann B 2008b Prognostic relevance of a novel TNM classification system for upper gastroenteropancreatic neuroendocrine tumors. Cancer 113 . (10.1002/cncr.23549) 18506737

[bib40] Rappel S Altendorf-Hofmann A Stolte M 1995 Prognosis of gastric carcinoid tumours. Digestion 56 . (10.1159/000201276) 8536814

[bib41] Ravizza D Fiori G Trovato C Fazio N Bonomo G Luca F Bodei L Pelosi G Tamayo D Crosta C 2007 Long-term endoscopic and clinical follow-up of untreated type 1 gastric neuroendocrine tumours. Digestive and Liver Disease 39 . (10.1016/j.dld.2007.01.018) 17433795

[bib45] Rindi G Luinetti O Cornaggia M Capella C Solcia E 1993 Three subtypes of gastric argyrophil carcinoid and the gastric neuroendocrine carcinoma: a clinicopathologic study. Gastroenterology 104 . (10.1016/0016-5085(93)90266-F) 7681798

[bib43] Rindi G Bordi C Rappel S La Rosa S Stolte M Solcia E 1996 Gastric carcinoids and neuroendocrine carcinomas: pathogenesis, pathology, and behavior. World Journal of Surgery 20 . (10.1007/s002689900026) 8661813

[bib42] Rindi G Azzoni C La Rosa S Klersy C Paolotti D Rappel S Stolte M Capella C Bordi C Solcia E 1999 ECL cell tumor and poorly differentiated endocrine carcinoma of the stomach: prognostic evaluation by pathological analysis. Gastroenterology 116 . (10.1016/S0016-5085(99)70174-5) 10029611

[bib44] Rindi G Kloppel G Alhman H Caplin M Couvelard A de Herder WW Erikssson B Falchetti A Falconi M Komminoth P, ***et al*** 2006 TNM staging of foregut (neuro)endocrine tumors: a consensus proposal including a grading system. Virchows Archiv 449 . (10.1007/s00428-006-0250-1) PMC1888719 16967267

[bib46] Rinke A Wiedenmann B Auernhammer C Bartenstein P Bartsch NB Faiss S Fottner C Gebauer B Goretzki P Jansen PL 2018 S2k Leitlinie Neuroendokrine Tumore. AWMF Register 021-26. Berlin, Germany: Arbeitsgemeinschaft der Wissenschaftlichen Medizinischen Fachgesellschaften e.V.

[bib47] Ruszniewski P Delle Fave G Cadiot G Komminoth P Chung D Kos-Kudla B Kianmanesh R Hochhauser D Arnold R Ahlman H, ***et al*** 2006 Well-differentiated gastric tumors/carcinomas. Neuroendocrinology 84 . (10.1159/000098007) 17312375

[bib48] Sagatun L Fossmark R Jianu CS Qvigstad G Nordrum IS Mjones P Waldum HL 2016 Follow-up of patients with ECL cell-derived tumours. Scandinavian Journal of Gastroenterology 51 . (10.3109/00365521.2016.1169588) 27309188

[bib49] Saund MS Al Natour RH Sharma AM Huang Q Boosalis VA Gold JS 2011 Tumor size and depth predict rate of lymph node metastasis and utilization of lymph node sampling in surgically managed gastric carcinoids. Annals of Surgical Oncology 18 . (10.1245/s10434-011-1652-0) 21455598

[bib50] Shen C Chen H Chen H Yin Y Han L Chen J Tang S Yin X Zhou Z Zhang B, ***et al*** 2016 Surgical treatment and prognosis of gastric neuroendocrine neoplasms: a single-center experience. BMC Gastroenterology 16 111 (10.1186/s12876-016-0505-5) 27613657 PMC5016962

[bib51] Solcia E Kloppel G Sobin L 2000 Histological Typing of Endocrine Tumours, World Health Organization International Histological Classification of Tumours. Berlin, Germany: Springer.

[bib52] Soto-Solis R Romano-Munive AF Santana de Anda K Barreto-Zuniga R 2019 Factors related to gastric neuroendocrine tumors. Revista de Gastroenterologia de Mexico 84 . (10.1016/j.rgmx.2018.03.002) 29705524

[bib53] Tang X Chen Y Guo L Zhang J Wang C 2015 Prognostic significance of metastatic lymph node number, ratio and station in gastric neuroendocrine carcinoma. Journal of Gastrointestinal Surgery 19 . (10.1007/s11605-014-2691-1) 25394386

[bib54] Thomas D Tsolakis AV Grozinsky-Glasberg S Fraenkel M Alexandraki K Sougioultzis S Gross DJ Kaltsas G 2013 Long-term follow-up of a large series of patients with type 1 gastric carcinoid tumors: data from a multicenter study. European Journal of Endocrinology 168 . (10.1530/EJE-12-0836) 23132699

[bib55] Uygun A Kadayifci A Polat Z Yilmaz K Gunal A Demir H Bagci S 2014 Long-term results of endoscopic resection for type I gastric neuroendocrine tumors. Journal of Surgical Oncology 109 . (10.1002/jso.23477) 24165913

[bib56] Vannella L Sbrozzi-Vanni A Lahner E Bordi C Pilozzi E Corleto VD Osborn JF Delle Fave G Annibale B 2011 Development of type I gastric carcinoid in patients with chronic atrophic gastritis. Alimentary Pharmacology and Therapeutics 33 . (10.1111/j.1365-2036.2011.04659.x) 21492197

[bib57] Vanoli A La Rosa S Miceli E Klersy C Maragliano R Capuano F Persichella A Martino M Inzani F Luinetti O, ***et al*** 2018 Prognostic evaluations tailored to specific gastric neuroendocrine neoplasms: analysis of 200 cases with extended follow-up. Neuroendocrinology 107 . (10.1159/000489902) 29895024

[bib58] Xie JW Lu J Lin JX Zheng CH Li P Wang JB Chen QY Cao LL Lin M Tu RH, ***et al*** 2017 Different long-term oncologic outcomes after radical surgical resection for neuroendocrine carcinoma and adenocarcinoma of the stomach. Oncotarget 8 . (10.18632/oncotarget.15488) PMC5593661 28915689

[bib59] Xu X Li J Han X Shi C Jin D Lou W 2014 Clinical characteristics and prognostic factors of patients with gastric neuroendocrine carcinoma treated with radical surgery. Chinese Medical Journal 127 . 24985576

[bib60] Yang Z Wang W Lu J Pan G Pan Z Chen Q Liu W Zhao Y 2018 Gastric neuroendocrine tumors (G-nets): incidence, prognosis and recent trend Toward improved survival. Cellular Physiology and Biochemistry 45 . (10.1159/000486915) 29402806

