# Supplementary material for: Gastric neuroendocrine neoplasias: manifestations and comparative outcomes
Source: Endocr Relat Cancer. 2019 Jul 4;26(9):751–63. doi: 10.1530/ERC-18-0582 (PMC6686747; doi:10.1530/ERC-18-0582)
Supplement: Supplemental Tables 1. Clinicopathologic characteristics of gNEN-1 according to resective treatment strategy Legend: ER endoscopic resection, EMR endoscopic mucosa resction, ESD endoscopic submucosa dissection, SR surgery, SD standard deviation, SEM standard error of mean, n number, BMI body mass in [file supplementary_table_1.pdf]

Felder et al. gNEN_Supplemental Tables

Supplemental Table 1.

	overall	ER (any)	SR	ER vs. SR
	**n (%)**	**mean ± SD (SEM), n**	**mean ± SD (SEM), n**	**P value ***
**n** (%)	86 (100)	76 (88.4)	10 (11.6)	n.a.
**Age,** years	85 (98.8)	56.91 ± 13.92 (1.60); 75	47.24 ± 7.86 (2.48); 10	***p=0.035***
**BMI** (kg/m^2)	58 (67.4)	26.00 ± 4.89 (0.69); 50	27.49 ± 7.17 (2.53); 8	p=0.472
**Amount**, numeric	51 (59.3)	4,13 ± 5.17 (0.771); 45	1,17 ± 0.40 (0.167) ; 6	***p=0,001***
**Size**,macro. (mm)	55 (63.9)	9,28 ± 7.55 (1.11); 46	19,77 ± 13.08 (4.36); 9	***p=0.044***
**Size**,micro. (mm)	59 (68.6)	7,26 ± 7.27 (1.01); 51	18,7 ± 15.60 (5.51); 9	***p=0.014***
**Ki67** (%)	65 (65.6)	2,51 ± 2.70 (0.35); 58	1,85 ± 1.21 (0.45); 7	p=0.528
**Gastrin** (pg/ml)	40 (46,5)	899,41 ± 621.09 (103.51); 36	1005.55 ± 681.01 (340.50); 4	p=0.749
**CgA** (U/l)	45 (52.3)	332.78 ± 337.56 (53.37); 40	11276.60 ± 24380.96 (10903.49); 5	p=0.372
