# Supplementary material for: Gastric neuroendocrine neoplasias: manifestations and comparative outcomes
Source: Endocr Relat Cancer. 2019 Jul 4;26(9):751–63. doi: 10.1530/ERC-18-0582 (PMC6686747; doi:10.1530/ERC-18-0582)
Supplement: Supplemental Table 2. Clinicopathologic Characteristics of patients with metastatic gNEN- 1 Legend: f female, m male, Age in years, ID initial diagnosis, FU follow up, H hepatic, LN lymphnode, EUS endoscopic ultrasound, SSR Somatostatin receptor, SSA Somatostatin Analoga, BII Billroth II, ER Endosco [file supplementary_table_2.pdf]

Felder et al. gNEN_Supplemental Tables

Supplemental Table 2.

patient	sex	age	time	tumor stage at time	Ki-67 (%)	size (mm)	amount	morphology	SSR Status	therapy	follow up (years)
1.	f	40.1	ID	pT2 p N1(1/3) Mx; IIIb	5	30	singular	central ulcerated	negative	S (BII, R0)	9.61
2.	m	32.5	ID	pT3pN1(8/11) pM1(H); IV	1	50	singular	central ulcerated	positive, Ga-68-Dota-toc, (H + LN)	S (lR1, R2) + SSA	1.66
3.	m	65	ID	pT4 Nx pM1 (H); IV	15	47	singular	smallcell	positive, SRS Octreoscan (H + Stom.)	PRRT (R2)	7.43 (dead)
4.	m	44.8	FU	T1 L1 Nx; IIIb	5	2	multiple	n.a.	n.a.	S (R0)	8.92
5.	m	53.2l	FU	pT2p N1 (1/17) pMx; IIIb	1	11	multiple	polypoid smallcell	negative, SRS Octreoscan (111-In-Octr.)	S (R0)	15.41
6.	m	55.3	FU	T2, cN1cM1 (H); IV	2	30	multiple	polypoid	positive, Ga-68-Dota-tate (H. + LN)	ER (lRx, R2)	4.37
